# Effect of acoustic standing waves on cellular viability and metabolic activity

**DOI:** 10.1038/s41598-020-65241-4

**Published:** 2020-05-22

**Authors:** Victoria Levario-Diaz, Pradeep Bhaskar, M. Carmen Galan, Adrian C. Barnes

**Affiliations:** 1 Bristol Centre for Functional Nanomaterials, HH Wills Physics Laboratory, University of Bristol, Bristol, BS8 1TL UK; 2 School of Chemistry, University of Bristol, Cantock’s Close, Bristol, BS8 1TS UK; 3 Department of Mechanical Engineering, University of Bristol, Bristol, BS8 1TR UK; 4 School of Physics, HH Wills Physics Laboratory, University of Bristol, Bristol, BS8 1TL UK

**Keywords:** Perturbations, Design, synthesis and processing, Acoustics

## Abstract

Acoustic standing wave devices offer excellent potential applications in biological sciences for drug delivery, cell manipulation and tissue engineering. However, concerns have been raised about possible destructive effects on cells due to the applied acoustic field, in addition to other produced secondary factors. Here, we report a systematic study employing a 1D resonant acoustic trapping device to evaluate the cell viability and cell metabolism for a healthy cell line (Human Dermal Fibroblasts, HDF) and a cervical cancer cell line (HeLa), as a function of time and voltages applied (4–10 V_*pp*_) under temperature-controlled conditions. We demonstrate that high cell viability can be achieved reliably when the device is operated at its minimum trapping voltage and tuned carefully to maximise the acoustic standing wave field at the cavity resonance. We found that cell viability and reductive metabolism for both cell lines are kept close to control levels at room temperature and at 34 °C after 15 minutes of acoustic exposure, while shorter acoustic exposures and small changes on temperature and voltages, had detrimental effects on cells. Our study highlights the importance of developing robust acoustic protocols where the operating mode of the acoustic device is well defined, characterized and its temperature carefully controlled, for the application of acoustic standing waves when using live cells and for potential clinical applications.

## Introduction

Ultrasound is a non-invasive, versatile and cost-effective tool that has been used for many years in a wide range of applications such as medical diagnostic imaging^1,2^, drug^1,3^ and gene delivery^1,4^. High intensity ultrasonic shock-wave methods are commonly used for breaking down kidney stones^5,6^, thrombolysis^5,7^ and physiotherapy^5,8^. Moreover, high intensity focused ultrasound (HIFU) is used for tumor treatment via localized hyperthermia or tissue ablation^5,9–11^. Recently, ultrasonic devices have been developed for cell manipulation for on-chip patterning^12,13^ where a well-defined spatial distribution is important for tissue engineering^14^ and developmental biology research^15^.

The ultrasound intensities used in clinical imaging diagnostics^16^ are comparable to those usually used to trap and manipulate particles in living cells and it is common to assume that there is a minimal effect on cell viability at these levels (up to 1 W/cm^2^ and 10 MHz). However, in studies involving cell viability under *in vitro* rather than *in vivo* conditions, concerns have been raised in particular for cells subjected to ultrasonic standing waves (rather than the progressive waves used for example, in imaging)^17^. Due to the application of ultrasound in medical applications, its effect on tissues has been studied extensively, not least to establish safe threshold limits^16,18^. However, fewer systematic studies have been done for *in vitro* applications using ultrasonic standing waves. To obtain sufficient trapping forces, standing wave devices must be used^19–21^. They may be produced from opposed transducers optimized to reduce the reflections at the operating frequency^22^ or by resonance reflections in a cavity where opposing walls are highly reflective. In the latter case, very high intensities in the hundreds of kHz to tens of MHz region may be produced by the multiple interference of the waves in the cavity^23^. The mechanisms by which ultrasound may cause cellular damage, depend strongly on frequency, intensity and nature of the field (progressive or standing wave) and have been recently discussed by Wiklund^17^. There are 4 main factors to consider when cells are subjected to ultrasound exposure: temperature, cavitation, acoustic streaming and radiation forces.

For instance, it is well known that living cells^24^ and tissues^25^ are very sensitive to changes in temperature. At moderate ultrasound intensities (~1 W cm^−2^) and frequencies (>1 MHz), similar to those used in typical manipulation devices, the direct generation of heat in the medium due to absorption is small^26,27^. However, the transducers and their mounting in any device may cause significant heating that may in some cases raise the temperature of the device by several degrees Celsius. This additional heat is difficult to regulate and, although some acoustic devices have achieved an optimal working temperature^28,29^, in most cases the uncontrolled extra heating can lead to significant death or damage to cells and tissues by hyperthermia^30^.

Ultrasound is also capable of causing cavitation (the formation and collapse of bubbles) in fluids^31^. These effects are difficult to quantify as they depend on the frequency and intensity of the ultrasound used and for instance the presence of dissolved gases in the fluid. The effect of ultrasound is proportional to $1/\sqrt{f}$ and hence it decreases at high frequencies and in the absence of dissolved gases is thought to be small for frequencies in excess of 1 MHz. In addition, for standing wave devices any bubbles formed by cavitation move to the pressure anti-nodes in contrast to the cells, that move to the nodes. As damage is greatest when the bubbles form close to the cells this separation will naturally reduce the damaging effect of cavitation^17^. Acoustic streaming (induced fluid flow due to the ultrasonic excitation) may also be produced in standing wave devices as demonstrated by Bassindale *et al*.^32^. It is known that strong streaming may be a source of cell damage, but on the other hand the reduction of temperature when the convective heat loss increases can also be beneficial^25,33^, as well as enhancing transdermal nanoparticle delivery^25,34,35^ and nonviral transfection to facilitate gene delivery^36^.

Cells are also subjected in a standing wave device, to oscillating pressures and gradients that give rise to acoustic radiation forces^37^. This can give rise to mechanical stress and loading of the cells and may in extreme cases lead to the destruction and fragmentation of the cells^38^. In addition to the effects of ultrasound, it is also important to consider any toxicity and contamination from the materials used to construct and assemble the device.

Several studies have reported that depending on the ultrasound frequency and intensity, cancer and healthy cells may behave differently^39,40^. While for healthy cells, i.e. Madin-Darby Canine Kidney (MDCK) cells, ultrasound seems to be stimulating cell proliferation and wound healing processes, in breast cancer cells, i.e. Michigan Cancer Foundation-7 (MCF-7) cells, apoptosis is induced suggesting they are more susceptible^39–42^. Although these mechanisms are not fully understood, previous work  has also demonstrated the rearrangement of the cytoskeleton and cell membrane damage in both cell types after ultrasound exposure^39,43,44^.

As stated above, despite the increasing use of acoustic waves in live cell applications, the effects of ultrasound on cell viability, metabolism and other biological parameters, are difficult to predict as it depends not only on cell type, but also on the type of ultrasound device, its mode of operation (applied voltages, frequencies, etc.), induced temperature changes and the effects of acoustic exposure and dose.

Herein, we present a systematic cell viability and metabolic study for two cell lines; a cervical cancer cell line (HeLa) and a healthy cell line (HDF) (Human Dermal Fibroblasts, HDF) in a 1D ultrasonic standing wave device using different voltages and exposure times. We address particularly the difficulties in using highly tuned resonant devices for studies of this type. We show that while cell alignment with acoustic trapping is easy to implement, when taking into account the different factors including type of device and temperature changes, getting a reliable correspondence between the pressure applied to the cells and the applied voltages and frequencies is not often possible due to the difficulty in dealing with what is a highly resonant system.

## Methods

### Cell culture

HeLa cells (passage 20) and human dermal fibroblasts (HDF, passage 7) were cultured in 75 cm^2^ flasks (Greiner Bio One) and maintained under standard cell culture conditions at 37 °C and with a 5% CO_2_ humidified atmosphere prior to experiments. Dulbecco’s Modified Eagle’s Medium (DMEM) with 4.5 g/L of D-Glucose (Gibco, Life Technologies, UK) was used for HeLa cells and Dulbecco’s Modified Eagle’s Medium (DMEM) with 1 g/L of D-Glucose used for HDF cells, both media supplemented with 10% FBS (Gibco, Life Technologies, UK) and 1% penicillin/streptomycin (Gibco, Life Technologies, UK) under sterile conditions.

### Acoustic device fabrication

**Figure 1:**
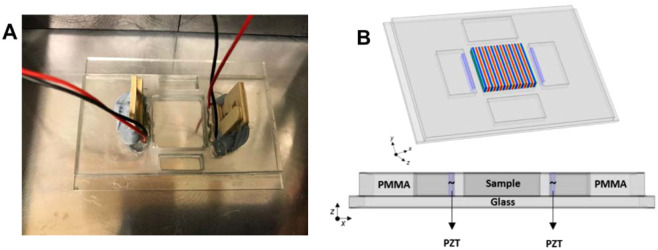
Diagram of the ultrasonic device. (**A**) Picture of the ultrasonic device where the opposed piezoelectric transducers (PZT) are wired and pressed to the PMMA wall with two wooden pieces. (**B**) Top: Representation of the 3-D view of the device; in the central chamber a schematic of the acoustic pressure field is shown. Bottom: a 2-D view of the materials employed in the ultrasonic device.

A schematic diagram of the acoustic trapping device is depicted in Fig. 1. A rectangular 2-D acoustic trapping device of the type described in previous works^32,45,46^ was manufactured and assembled with two front-faced 1 mm thick piezoelectric transducers (PZT) (Noliac, NCE51, L 15 X W 2 mm). Trapping was only carried out in 1D so that the cells aligned in rows parallel to the transducer faces. The acoustic device was fabricated from PMMA [Poly(methyl methacrylate)] and mounted on a double-width glass slide (Corning, L 75 X W 50 mm). The PZT transducers were positioned in the adjacent cavities coupled with ultrasound gel (Sonatest W250) pressed to the device wall by two wooden pieces, acting as springs, separated by a 3 mm boundary from the central cavity. This wall ensures there is no physical or electrical contact of the transducers with the trapping medium to prevent any unwanted contamination. The width of the cavity was *w*~20 mm. The transducers were operated close to their third harmonic frequency (6.74 MHz) that was determined experimentally by electrical impedance measurements. The parallel opposed transducers were driven close to their antiresonance frequency (~6.77 MHz), with a corresponding wavelength of λ = 220 µm (in water). It is important to observe that small changes in temperature (1–2 °C) are sufficient to cause loss of resonance condition. The device was operated at a cavity resonance given by *w*/λ is integer. These resonances are separated by ~0.1 MHz and the frequency was adjusted (in steps of 0.01 MHz) each time until cell alignment was observed. The counter-propagating waves were generated with a single signal generator (GwInstek SFG-2020) in parallel and monitored with an oscilloscope (Agilent Technologies DS03152A). When driven at 10V_*pp*_ the maximum electrical power delivered to the device is ~1 W.

### Cell alignment rate deposition

Square glass coverslips (18 × 18 mm) were coated with 0.01% poly-L-lysine (A-005-C, Merck Millipore) overnight and washed with PBS 1× (pH 7.4, Gibco, Life Technologies, UK) prior to the experiments. A coated-glass coverslip was positioned in the central chamber where 650 µl of cells, after being trypsinized from confluent cultures, were seeded in the central chamber of the acoustic trap at a concentration of 5 × 10^4^ cells ml^−1^, which was measured employing a dye-exclusion assay and with an automated cell counter. In order to induce cell trapping at the bottom of the device, two deposition methods were then used: firstly the acoustic field was applied immediately after the cells were seeded in the chamber (e.g. trapping as the cells settled) and secondly, the cells were allowed to settled for three minutes before the field was applied (e.g. trapping after the cells had deposited).

The standing waves generated in the cavity guided the cells to the acoustic pressure nodes, where the trapping and aligning occurred. After 5, 10 and 15 min of acoustic exposure, the coverslips with the aligned HeLa and HDF cells, were placed in a 6-well plate for 24 h incubation with the respective DMEM medium. Pictures and videos showing the cellular deposition over time were obtained using a Leica DMIL inverted microscope with a mounted Leica DFC295 digital camera and further analyzed with LAS-MultiTime-Movie-Timelapse (Leica Microsystems Limited, UK). See ESI for full details.

### Temperature-dependent measurements

Temperature measurements were made in the central chamber filled with biological media (where the cellular trapping occurs) for different applied voltages in the range (4–10 V_*pp*_) to study temperature changes in relation to exposure time and intensities. These voltages are comparable to those used for live cell assays with potential biomedical applications according to previous literature^12^. The acoustic device was then placed in a block heater (digital block heater SBH130D, Stuart, Cole-Parmer, UK), to keep the temperature stable (34 °C) and the temperature variations were then compared to those measured when testing the same voltages in the device kept at room temperature (20 °C) without temperature control. The central chamber was filled with 650 µl of DMEM media and temperature recordings were taken every minute for a total of 30 min while the acoustic field was switched on. Measurements were monitored using a Type K thermocouple (Fisher Scientific, UK) and repeated three times.

### Cell viability and metabolic activity

Following the initial experimental calibrations described above, cell viability and metabolic activity assays were performed for HeLa and HDF cells acoustically exposed to 6, 8 and 10 V_*pp*_ for 5, 10 and 15 min after a 24 h incubation. As a control, cells without acoustic exposure were maintained at the same temperature and for the same amount of time in the device cavity as the exposed cells.

Cell metabolism was assessed using 5% v/v AlamarBlue (560_Ex_/590_Em_) (Invitrogen, UK) in DMEM medium without FBS. AlamarBlue is a cytosolic substrate for reductive enzymes (resazurin to resorufin) whose fluorescence spectrum changes on reduction. The total number of live cells was evaluated using 3 µM Calcein-AM (490_Ex_/520_Em_) (Invitrogen, UK), which is transformed into fluorescent Calcein in the cytoplasm of live cells. HeLa and HDF cells were washed with PBS 1X prior incubation at 37 °C for 1 h, with both dyes simultaneously. Fluorescence emissions were measured for exposed and control cells, collected from 50 µl of cell culture supernatant and acquired with the CLARIOstar® microplate reader (BMG LABTECH, UK). Readings were repeated three times.

## Results and Discussion

### Cell trapping vs rate deposition

**Figure 2:**
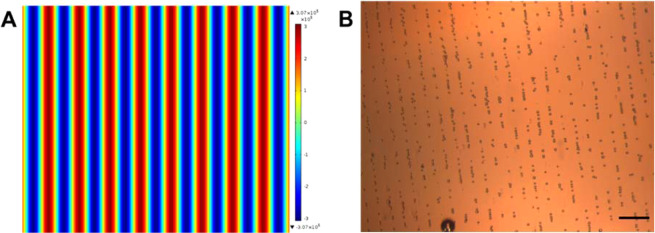
(**A**) A schematic of the acoustic pressure in the device for the expected cellular patterning with two active piezoelectric transducers (PZT); pressure nodes depicted in red and anti-pressure nodes in blue^8^ (**B**) HeLa cells in a linear patterning, acoustically trapped in the pressure nodes of the standing wave. Scale bar 100 µm and objective 4× magnitude.

HeLa cells (diameter ~ 12 µm) were used for cellular deposition and acoustic patterning evaluations. The cell alignment observed agrees with model predictions when the two opposed transducers are driven at the same frequency (6.77 MHz) and results in parallel lines of aligned cells separated by 110 µm (see Fig. 2). The linear patterning is achieved as a result of the standing waves generated by multiple reflections (resonance) from the ultrasonic waves reflected in the device.

Scholz, *et al*. has demonstrated^13^ that the acoustic patterning of various spherical and cylindrical microparticles was generated near the bottom surface of the device due to the high force distribution. To better define the optimum parameters for acoustic trapping of live cells and to help predict their behaviour under ultrasonic conditions, deposition studies were thus carried out. To that end, the rate of cell deposition without any external input was determined to be 3 min. (See video of standard cellular deposition in Supplementary Information).

**Figure 3:**
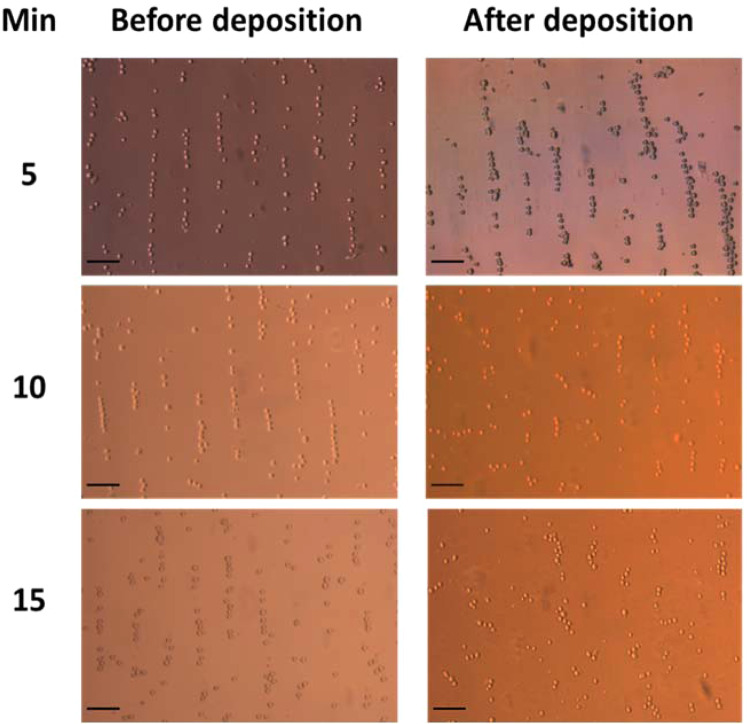
HeLa linear alignment patterning after acoustic trapping before and after cells being deposited at the bottom of the acoustic device at three different times (5, 10, 15 min). Objective 10× magnitude and scale bar 50 µm.

The HeLa cell patterning and bottom surface attachment were evaluated at an amplitude of 6 V_*pp*_ at 5, 10 and 15 min of acoustic exposure. Optical microscope images were acquired throughout the alignment demonstrating that cell trapping, and linear patterning were achieved under both deposition procedures at the three times tested (Fig. 3) and that cells were mostly intact. However, at 10 and 15 min, allowing the cells to deposit before applying the acoustic field, led to a more diffuse patterning, and less well-defined cell alignment. These observations suggest that cell adhesion to the poly-l-lysine treated glass coverslip taking place as the cells deposit at the bottom of the device is difficult to overcome with the weaker acoustic forces once the cells have attached. Indeed, previous work employing a similar device, whereby the forces exerted onto silica micro-beads under acoustic standing waves were measured with optical tweezers under a range of voltages (2–6 V_*pp*_) showed these forces to be in the in the order of 0.5–1 pN V^−1^ depending on the amplitude of the voltage applied^32^. While on the other hand, Touhami *et al*.^47^ has reported that adhesion forces of poly-l-lysine to bacterial cells are typically greater than a few hundreds of pN. Moreover, our observations suggest that the acoustic forces achieved with our device should be considerably less than the ~ 100 pN that are known to cause cell rupture^48^.

**Figure 4:**
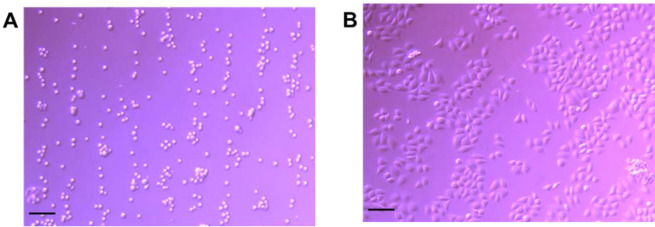
Aligned HeLa cells at 6 V_*pp*_ at 5 min of acoustic exposure at rt. (**A**) Linear patterning of HeLa cells after seeding in the central cavity and (**B**) maintained linear patterning after a 24 h incubation. Scale bar 100 µm and objective 10× magnitude.

It was thus decided that for optimum linear arrangement it is necessary to switch on the acoustic field before adding the cells into the central cavity. Under these deposition conditions, there was no difference in the patterning or number of attached cells observed at the three exposure times tested. This is an important consideration for single cell analysis or tissue engineering^14^ type-studies. It is important to note that after a 24 h incubation, the cell linear patterning and orientation were maintained, as shown in Fig. 4.

**Figure 5:**
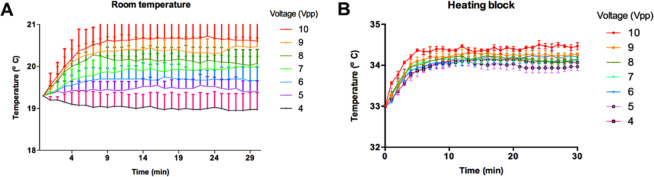
Temperature monitored in the central chamber of the ultrasonic device at (**A**) room temperature (~20 °C) and (**B**) temperature-controlled stage set at 34 °C throughout 30 min at different voltages (4–10 V_*pp*_).

As discussed previously, it is known that the temperature of the fluid in the device has an impact on cell viability and metabolism. We hence carried out a careful measurement of the temperature of the media in the device when operated at room temperature (20 °C without temperature-control) and also when the temperature is kept at 34 °C. Figure 5A shows the temperature of the fluid in the device as a function of time for the different driving voltages with the measurements starting at ambient temperature. A rise in temperature of ~2 °C was observed at the highest driving voltage (10 V_*pp*_) with an equilibration time of about 10 minutes. This is consistent with the total electrical output power of the device and its thermal mass. In contrast, when the cell was initially heated to 33 °C (Fig. 5B) a final temperature of 34 °C was recorded by the thermocouple in the central cavity with fluctuations of less than 0.25 °C irrespective of the driving voltage. This temperature was chosen because it is close to physiological conditions and cell health of untreated controls remain high. It should be noted that small changes in temperature will change the acoustic resonance frequency of the cavity and the frequency was periodically adjusted to optimise trapping.

### Cancer and non-cancer cell viability and metabolic activity

After optimization of the conditions required for cell deposition, we evaluated the impact of ultrasound on a cancer cell line (HeLa) and a healthy cell line (HDF) at three different times (5, 10 and 15 minutes) and voltages (6, 8 and 10 V_*pp*_). It is important to note that in our device, alignments carried out under the same conditions and voltages for 30 min or 1 h lead to cell death. A similar effect was observed when using voltages higher than 10 V_*pp*_, even at short reaction times, and at lower than 6 V_*pp*_, cell alignment was not observed. For this reason, we decided to center the temperature study on the optimum parameters.

**Figure 6:**
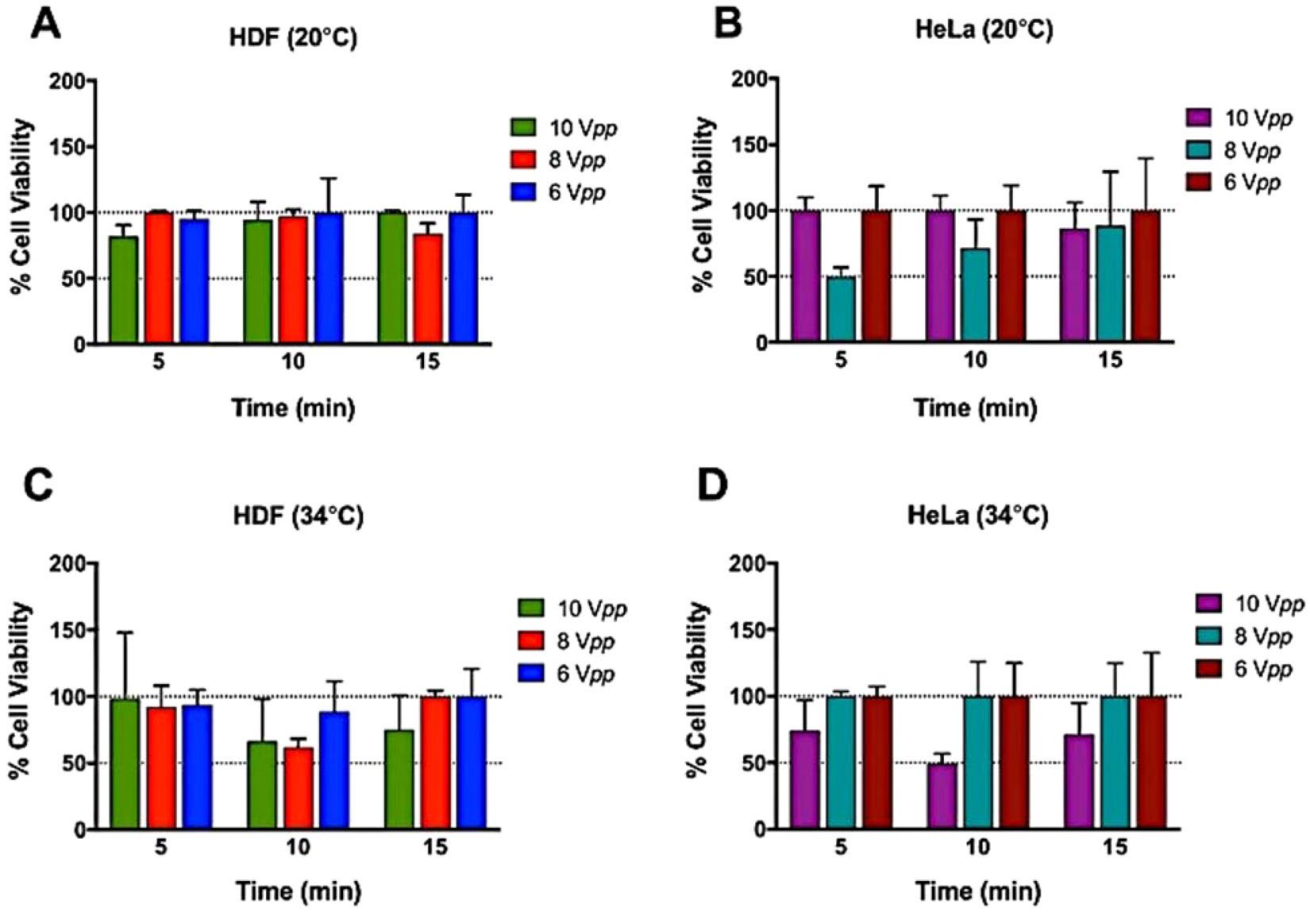
HDF and HeLa viability graphs after acoustic exposure relative to 100% controls. (**A**) and (**B**) HDF and HeLa evaluated when at 20 °C, respectively, and (**C**) HDF and (**D**) HeLa when kept at 34 °C with a heating block. Data shown as mean ± SD, per triplicate. The results are given as percentage values referenced to untreated controls (100%).

Prior to the experiments, HeLa and HDF cell viability was measured employing a dye-exclusion assay and with an automated cell counter indicating a 97% and 98% cell viability, respectively. As mentioned before, cell viability of exposed and control samples was evaluated using a Calcein-AM assay. The results (Fig. 6A) show that HDF has a good viability for all voltages and times when carried out at room temperature. In contrast, when the temperature was set to 34 °C (Fig. 6C) prior to alignment, cell death increased after 10 min of acoustic exposure, particularly at 8 and 10 V_*pp*_ where the viability decreased to near ~50% with respect to untreated controls. On the other hand, HeLa cells (Fig. 6B) did not behave as well at room temperature and viability decreased to 50% after just 5 min acoustic exposure compare to the control when the driving voltage was 8 V_*pp*_ and remained low after 10 min exposure. Meanwhile, at driving voltages of either 6 or 10 V_*pp*_ the viability remained high (>80%) for all exposure times at room temperature. When the device was maintained at 34 °C (Fig. 6D), cell health was maintained except at the highest voltage (10 V_*pp*_ after 10 min). Interestingly, it was generally observed that cell health was maintained at 8 and 6 V_*pp*_. However, a significant decrease in viability was noted when the driving voltage was set at 10 V_*pp*_ after a 10 min acoustic exposure while at 5 and 15 min the cell viability was maintained.

**Figure 7:**
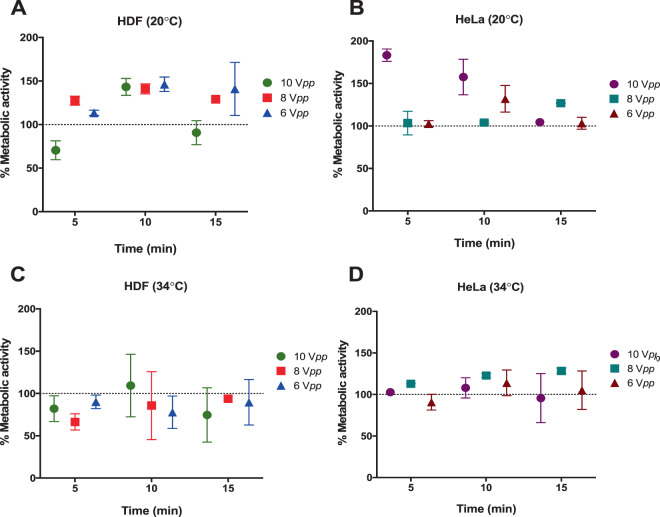
Reductive metabolism of HDF and HeLa after acoustic exposure relative to 100% controls. Alamar Blue assays were conducted at three time points (5, 10 and 15 min) at 10, 8 and 6 V_*pp*_. (**A**) HDF and (**B**) HeLa maintained at room temperature (20 °C) and (**C**) HDF and (**D**) HeLa maintain the temperature at 34 °C. Data shown as mean ± SD, per triplicate. The results are given as percentage values referenced to untreated controls (100%).

Metabolic activity, which is an indication of cytotoxicity, after the ultrasound exposure in the trapping device for HDF and HeLa cells was measured and compared to unaligned control samples (Fig. 7). In general, minimal or no significant changes on the metabolic rate for both cell lines kept at 34 °C under ultrasonic exposure at the three different voltages were observed. In studies carried out at room temperature without temperature control, the metabolic activity in the acoustically exposed samples differed depending on cell line. Changes in metabolic activity were detected for HDF and HeLa cells after 5 and 10 minutes at an amplitude of 10 V_*pp*_. Metabolic rate appears to be maintained at the highest amplitudes after 15 mins of continued acoustic exposure, as previously observed for cell viability, suggesting cells can recover from the initial acoustic shock after a minimum period of adjustment.

Our results demonstrate that different cell types are affected differently to ultrasound voltage exposure, time and temperature combinations. This is not unexpected as cell membrane biophysical properties differ between cell lines and changes in temperature also influence cell stiffness^39^. Moreover, it has been proposed that those differences could be exploited to mechanically discriminate between tumour and normal cells^39^. However, a clear correlation between the applied voltage, exposure time, the dose and temperature are not observed for both the HDF and HeLa cells in our experiments. As discussed earlier, there are several factors that may have a detrimental impact on cell health (e.g. temperature, cavitation, acoustic stress, streaming or from the materials used in the device) that might affect cells differently. We believe that the variations of these factors may be the origin of the inconsistencies that we observe in the cell viability.

At the frequencies used in this device, we do not expect strong localised heating around the cells as previously discussed in Wilkund^17^. At room temperature and in the absence of any temperature control, the operating temperature of the device increased only slightly as the driving voltage increased (maximum 2 °C at 10 V_*pp*_ when the device reached equilibrium) and no changes in temperature were detected when the device was actively controlled at close to physiological temperatures of 34 °C ± 0.2 °C, we can therefore conclude that the observed changes in viability are not just as a result of overheating.

Cavitation effects are expected to be weak at the frequencies used and should increase as the driving voltage is increasing^31^. Furthermore, during the measurements no evidence of bubble formation, or migration of bubbles was observed. We thus conclude that our results are not explained by any obvious cavitation. On the other hand, devices of this type are known to cause streaming of the fluid. We would, however, expect the effects of streaming to increase with the applied voltage^32^ and we believe it is unlikely that streaming is the cause of the variation in cell viability that we observe.

As noted earlier, this is a standing wave device in which a stationary acoustic wave is created within the device. In operation, the cells will be directed to the pressure nodes in the standing wave field produced by the transducers. In the device used here, two opposed piezoelectric transducers, located behind PMMA walls (to avoid contact between the cells and transducers), are used to produce the standing wave. We note that unless the transducers have been designed to minimize their reflectivity at the operating frequency (by use of quarter wave coatings or by working strictly at resonance for perfectly matched transducers^22^) the standing wave field is dominated by multiple reflections at the transducer faces – that is by acoustic resonance in the cavity.

In this work the transducers were operated away from their resonance (closer to anti-resonance) such that these cavity resonances will dominate the trapping field. A thorough experimental and theoretical finite element analysis of the operation of this type of device was given by Scholz *et al*.^13^. Their results demonstrate the existence of the standing wave field but also show that it is confined to a thin layer above the bottom plate. Their electrical impedance measurements show a strong correlation with the forces derived from the finite element analysis and the peak acoustic pressures observed. Of note is the highest pressures are observed away from the transducer resonances and are closer to their antiresonance as we found. In addition, large variations in pressure over a narrow frequency range, characteristic of the cavity resonances, are observed clearly. A simple and important conclusion from their work is that large variations (an order of magnitude) in the acoustic pressure may occur for very small changes in frequency, temperature (as the position of the resonances depends on the speed of sound in the medium) and other experimental parameters such as the acoustic coupling between the transducers and the device.

**Figure 8:**
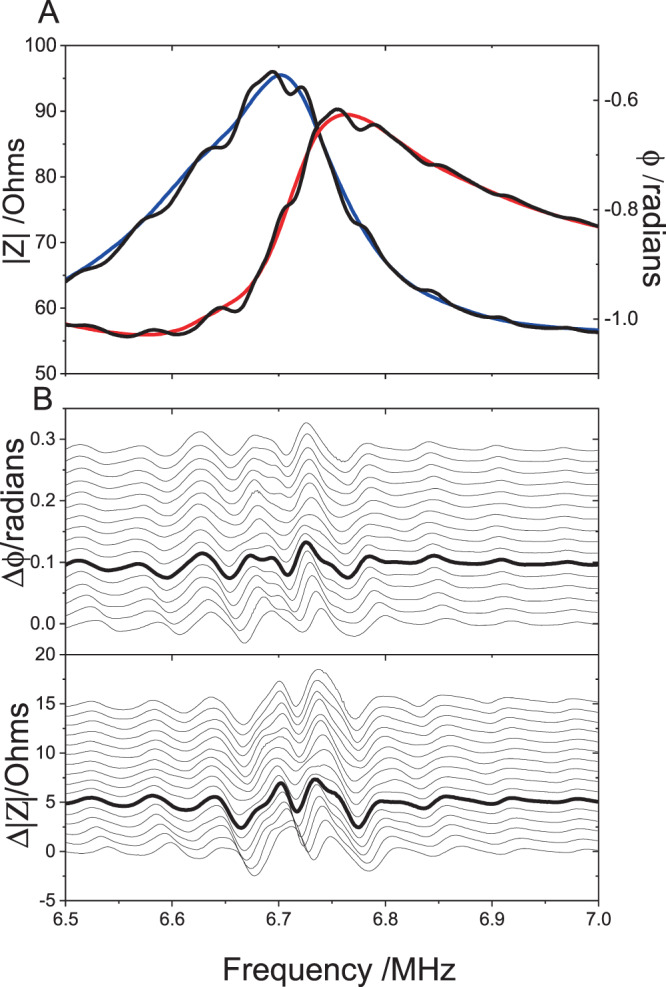
(**A**) The electrical impedance |Z | (red line) and phase angle φ (blue line) for the two transducers connected in parallel for the device before filling. The black lines show the corresponding impedance when water is added to the cell. The modulation of the impedance with a period of ~60 kHz, due to cavity resonance, is observed clearly. (**B**) The changes in the modulation seen in the impedance measurements as the volume of water is increased successively in 20 µL steps. The lowest trace corresponds to the underfilled device. The bold line corresponds to a level surface in the device as used in our measurements. For clarity the smooth unfilled cell impedance (red and blue lines in (**A**) has been subtracted and the measurements offset by +0.05Ω ( | Z | ) and +0.001 radians (φ) for each step. The large variations in the impedance close to the operating frequency of the device (6.77 MHz) can be observed.

In order to investigate these effects in our device we have carried out detailed electrical impedance measurements under the typical experimental conditions we used. As shown in Fig. 8A, in the absence of fluid in the device, the electrical impedance of the device is in accordance to what is expected when two transducers of slightly different resonance frequencies are connected in parallel. When water is added to the cavity a clear modulation of the impedance is observed with a frequency separation of ~60 kHz as expected from the length of the cavity (fluid and PMMA walls). This supports the conclusion that the device is working at a strong cavity resonance as observed by Scholz *et al*.^13^. Hence, in our device considerable variations in the acoustic amplitude and stress on the cells may occur for very small changes in frequency (much less than the 10 kHz steps used to obtain alignment). Furthermore, although, in theory, the maximum pressure in the device is expected to be proportional to the applied transducer voltage, the large variations in pressure (at constant applied voltage) with subtle changes in frequency mean that the voltage applied to the device is not a reliable indication of the maximum acoustic pressure applied to the cells within it.

It can also be noted that the wavelength of the cavity resonance must be *nλ* = *w* or, in terms of frequency, *v* = *nv*/*w* where *v* is the velocity of sound. At room temperature the velocity of sound in water is ~1500 m s^−1^ but changes at a rate of ~3 m s^−1^ °C^−1^. Hence, a small temperature change of 1 °C will shift the resonance by ~0.01 MHz to move away from the optimum resonance condition. Therefore, in practise it is difficult, during a measurement, to establish the precise acoustic intensity for each exposure or within the same exposure if the temperature is changing with time.

We have also considered differences in acoustic pressures that may arise due to other variations in the experimental conditions, especially the filling of the device. In the calculations of Scholz *et al*.^13^ it is assumed that the liquid is level with the top of the devices (with no meniscus). However, given the depth variation in the fringe intensities they observe it is plausible that the acoustic pressures at the bottom of the device may also depend on the effect of reflections from the top surface (and hence its height). Figure 8B shows the variation in the resonance fringes in the impedance measurements as the cell is filled from slightly under-full to slightly overfull in steps of 20μL. While variations away from the transducer resonances (<6.6 MHz and >6.8 MHz) are small, there are noticeable changes around the frequency (6.77 MHz) where we found strongest alignment of cells, indicating that the acoustic pressure on the cells is also sensitive to the filling of the device.

In a last test, the device was emptied with a syringe and left undisturbed overnight. It was refilled the following morning without movement or further disturbance and the impedance measurements were repeated. No evidence of any modulation in the impedance was observed. When the device was dismantled it was clear that the ultrasound gel had dried out such that there was very poor acoustic coupling between the transducers and the device. After cleaning and re-assembling the device with new gel it performed as before.

The difficulty in defining the precise operational conditions of the device appears to be the origin of the inconsistent results we observe, and that other authors report for cell viability. In practice, it is not possible to tune accurately these devices to be precisely at resonance and even if this is possible, small changes in temperature are sufficient to cause substantial changes to the acoustic forces applied to the cells. We cannot assume that the acoustic pressure applied to the cells is proportional to the voltage applied to the device. Therefore, we strongly encourage that care should be taken to apply only the lowest voltages needed for trapping. The optimal trapping should then be achieved by subtle tuning the frequency (in steps much less than 0.01 MHz) without increasing the applied voltage. Indeed, we have found in our device that with this procedure, successful trapping may be obtained reliably at 4 V_*pp*_. It is also important that the effect of the acoustic properties of the material (glass, PMMA, etc.) used to construct the device are considered when operating for an optimum condition.

## Conclusions

In summary, we have used a resonant ultrasonic trapping device to study what are the key parameters that affect viability and metabolic activity of two different cell lines (epithelial and cancer) at room temperature and 34 °C. We found that cell viability was not only dependent on the device specifications (voltage, temperature, exposure time), but also on cell type. Indeed, high mortality of HeLa cells was observed at 8 V_*pp*_ after 5 and 10 minutes of exposure at 20 °C, while HDF cells were unaffected under the same conditions. As mentioned before, small changes in temperature affected cell viability and metabolic activity differently, which makes it hard to predict which cell type will respond better. We conclude that the inconsistencies in cell profiles that we observe may be attributed to the difficulties in defining precisely the acoustic intensities in highly resonant devices of this type. We find, that by developing robust protocols where the minimum applied voltages is used, followed by careful tuning of the resonant cavity, cells can be trapped reliably, while maintaining high cell viability. We observe that in our experimental set up, 15-minute ultrasound exposure is optimum, particularly for HeLa cells in contrast to HDF cells. Decoupling temperature effects from ultrasound effects would be difficult, however, from the impedance measurements carried out under different experimental conditions (see ESI), it is clear that small variations on the acoustic parameters have significant effects on the acoustic pressures. Nevertheless, one cannot completely dismiss temperature effects alone, since thermorheology changes have been also reported to have an impact on cell mechanics, which in turn will affect cell health^49^.

While we find that performing ultrasonic alignment at colder temperatures (20–22 °C) appears better in general for cell viability (albeit to the detriment of metabolic activity which is increased and might be a sign of cytotoxicity), the device parameters can be tuned to favor general cell health at 34 °C which is closer to physiological conditions.

Based on our evidence, if ultrasound is to be employed in clinical diagnosis or in a therapeutic setting, it is important to carry out systematic cell trapping/manipulation studies under physiological conditions, since small changes in temperature have a significant effect on acoustic pressures and in turn will affect cell stress and ultimately cytotoxicity, which is also highly dependent on cell type. Refinement of acoustic experimental protocols and measurements of the actual changes in acoustic intensity occurring in the device, as a function of temperature and frequency under typical operating conditions, are key as we have found.

## Supplementary information

Supplementary information.

## Data Availability

All underlying data are provided within this manuscript and in its supplementary information file. The supplementary information file includes information about the acoustic resonant cavity, electrical impedance models and comparison to experimental measurements and calculations for cellular concentrations.
