# Supplementary material for: Effect of acoustic standing waves on cellular viability and metabolic activity
Source: Sci Rep. 2020 May 22;10:8493. doi: 10.1038/s41598-020-65241-4 (PMC7244593; doi:10.1038/s41598-020-65241-4)
Supplement: Supplementary file 1 — Supplementary information. [file 41598_2020_65241_MOESM1_ESM.docx]

**Supplementary information for:**

**Effect of acoustic standing waves on cellular viability and metabolic activity**

Victoria Levario-Diaz,^1,2^ Pradeep Bhaskar,^3^ M. Carmen Galan *^2^ and Adrian C. Barnes *^4^

^1^ Bristol Centre for Functional Nanomaterials, HH Wills Physics Laboratory, University of Bristol, Bristol BS8 1TL, UK

^2^ School of Chemistry, University of Bristol, Cantock’s Close, Bristol BS8 1TS, UK

^3^ Department of Mechanical Engineering, University of Bristol, Bristol BS8 1TR, UK

^4^ School of Physics, HH Wills Physics Laboratory, University of Bristol, Bristol BS8 1TL, UK

**Background.**

The details of the principle of operation of acoustic trapping devices working at these frequencies was detailed in the work of Courtney et. al.^1^, especially with regard to particle *manipulation* (translation in space by independently changing the relative phase of the transducers). To meet the conditions required for manipulation, the transducers need to be transparent at their operating frequency. This can be achieved by the application of a quarter wave anti-reflection coating or, operating the transducers precisely at their resonance frequency where the reflection becomes close to zero. The latter requires very close frequency matching of the transducers, as the reflection coefficient away from resonance rises rapidly from zero to close to one. Our device used relatively poorly matched transducers without anti-reflection coatings, so it is hard to avoid reflections in the cavity.

If the aim of an experiment is to achieve particle trapping, as opposed to trapping *and* manipulation the stringent matching of the transducers may be relaxed, and higher trapping forces may be achieved by exploiting the cavity resonances cause by multiple reflections between the transducers. In the work reported here the resonant frequency (third harmonic) of the transducers used were 6.69 MHz and 6.75 MHz respectively and strong effects from the cavity resonances were observed as described below.

**An acoustic resonant cavity.**

The acoustic pressure as a function of position in a one-dimensional cavity may be represented most simply in terms of the acoustical equivalent of the optical Fabry-Perot Cavity.

$$P\sim P_{0}\frac{1}{\left( 1-\sqrt{r_{1}r_{2}e^{-\alpha f^{2}d}} \right)^{2}+4\sqrt{r_{1}r_{2}}e^{-\alpha f^{2}d}\sin^{2} (\frac{\pi d}{\lambda})}$$

where $r_{1}$ and $r_{2}$ are the reflection coefficients of the walls, $\alpha$ is the linear attenuation coefficient of the wave, $f$ is the frequency of the acoustic wave, $d$ is the length of the cavity and $\lambda$ is the wavelength. Figure S1 below shows the maximum pressure, in arbitrary units, obtained in a cavity as a function of frequency, for different values of $r=r_{1}=r_{2}$. In a typical device, away from the resonance frequency of the transducers $r$ is typically of the order 0.8-0.9 so it can be seen that quite substantial changes in the peak pressure will be obtained for quite small changes in frequency. Due to the change in the velocity of sound in the fluid, that is dependent on temperature, the frequency for the maximum pressure is also sensitive to the temperature of the devices and any other small changes in its operational parameters.


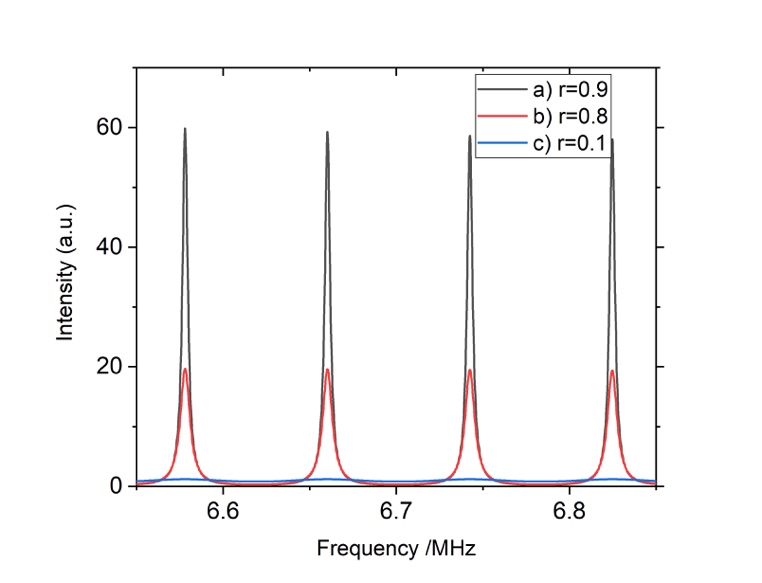


**Figure S1**. The maximum intensity as a function of frequency of the acoustic standing wave in a cavity of length 18mm containing water: a) for a reflection coefficient r=0.9 similar to that of PZT walls away from the transducer resonance, b) for r = 0.8 corresponding to walls of silica and c) for r = 0.1 corresponding to walls of PMMA

In practise, in any typical experiment it is difficult to determine and maintain the device precisely at a cavity resonance meaning will be a large uncertainty in the relative pressure that will be applied to a particle in a trap. In particular, small changes in applied voltage, temperature or frequency will give rise to large pressure changes.

As the device will absorb more power at a cavity resonance (as with any other resonance) we will expect to see characteristic changes in the electrical properties of the device as we pass through resonance.

**Electrical Impedance models.**

The resonant character of the device is most easily observed by measuring its electrical impedance as a function of frequency. In the absence of any acoustic resonances the impedance should correspond to that of two independent transducers connected in parallel. Figure S2 shows an impedance calculation, using a simple (1 dimensional) transmission line model in SPICE for the two PZT transducers with resonant frequencies set at 6.69 MHz and 6.74 MHz (as used in the experiments). This corresponds well with the electrical impedance measurement we made for our empty device.


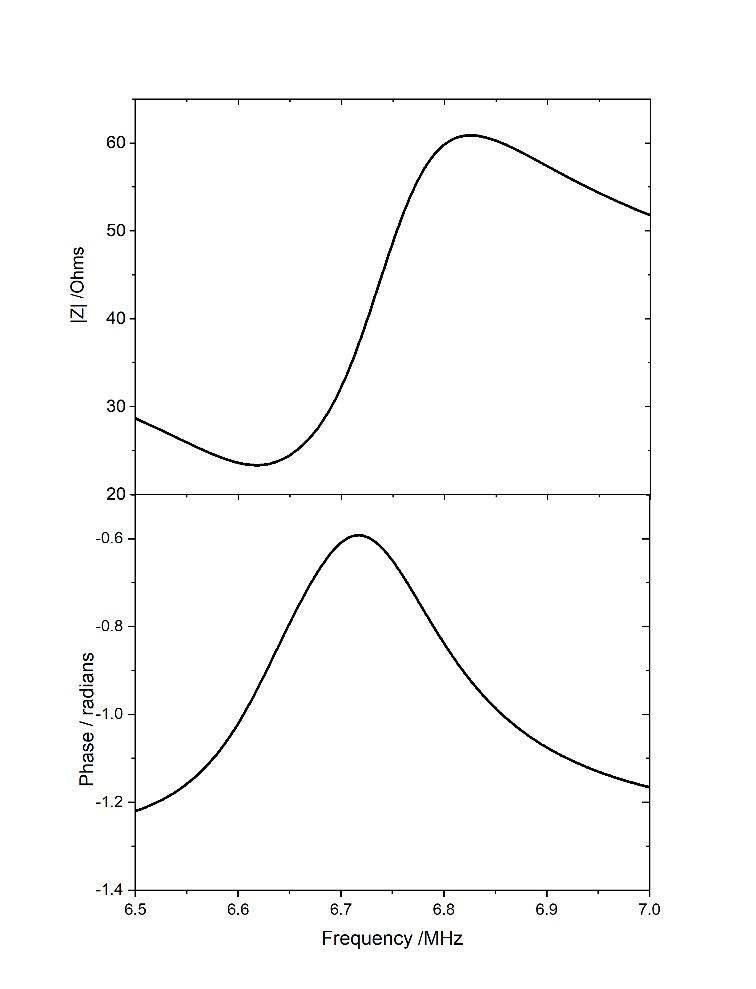


**Figure S2**. Impedance calculated with a transmission line model for two piezoelectric transducers in the device with no fluid in the cavity. This may be compared with the measurements shown in figure 8 (the red and blue curves).

Figure S3 shows the same calculation when the transmission line model includes the effects of a cavity with 20mm of water and PMMA walls of 2.5 mm between the transducers representing the configuration of the experimental device.


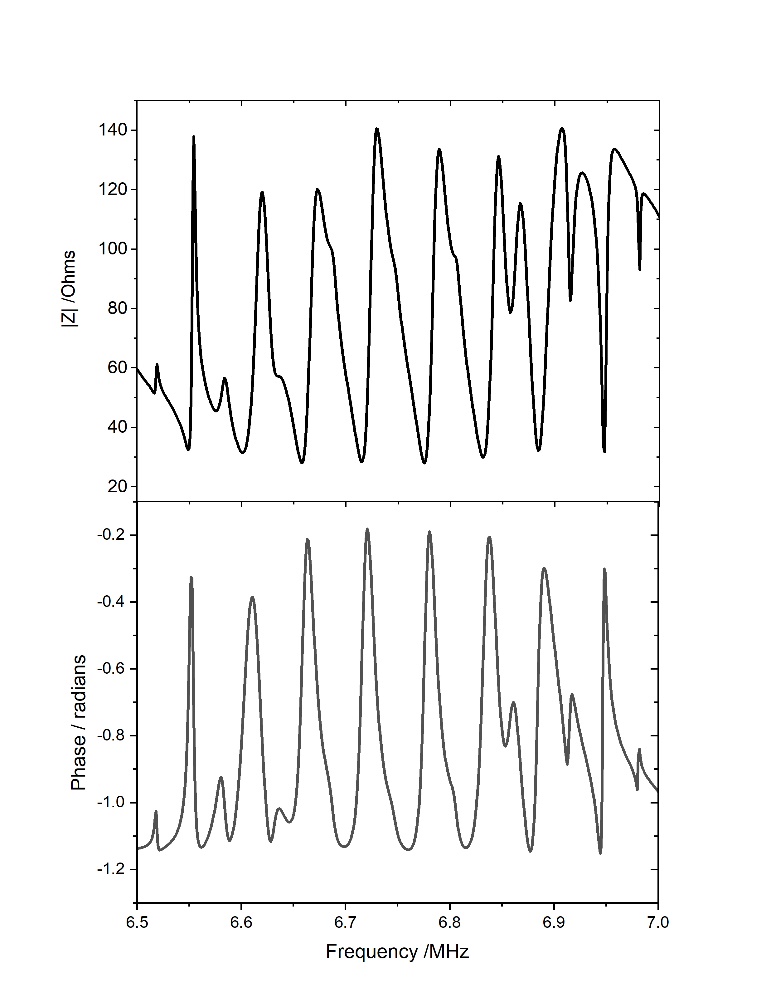


**Figure S3**. A transmission line calculation for the impedance of the device with 2.5mm PMMA walls and 20mm water in the cavity.

This calculation shows the effect of the highly resonant cavity on the electrical impedance. The main spacing (~60 kHz) of the dips in |Z| and the peaks in ⱷ corresponds to the cavity resonances characterised by the separation of the transducer crystals. The smaller features correspond to weaker interference effects due to reflections between the PMMA walls and the rear faces of the transducers. This is the result we would expect for impedance measurements if the *whole* of the cavity was in resonance. In our and in previous measurements (see for example Scholz et. al (reference 13, figure 7) such strong effects have not been observed. This observation is consistent with the result reported by Scholz et. al. (reference 13, figure 6), from a Finite Element Analysis, that only a small volume of the cavity, close to its base shows this strong resonant cavity behaviour.

Recognising that only a small volume of the cavity will be actively resonating we have modified the transmission line model by considering the device as two separate parts connected in parallel, a non-resonant part acting in a similar way to that shown in figure S1 and a resonant part similar to that shown in figure S2. Figure S4 shows the impedance calculation from this model when the fraction of the radiating surface of the transducers in the resonant part of the chamber, compared to that in the non-resonant chamber was set as 1/20.


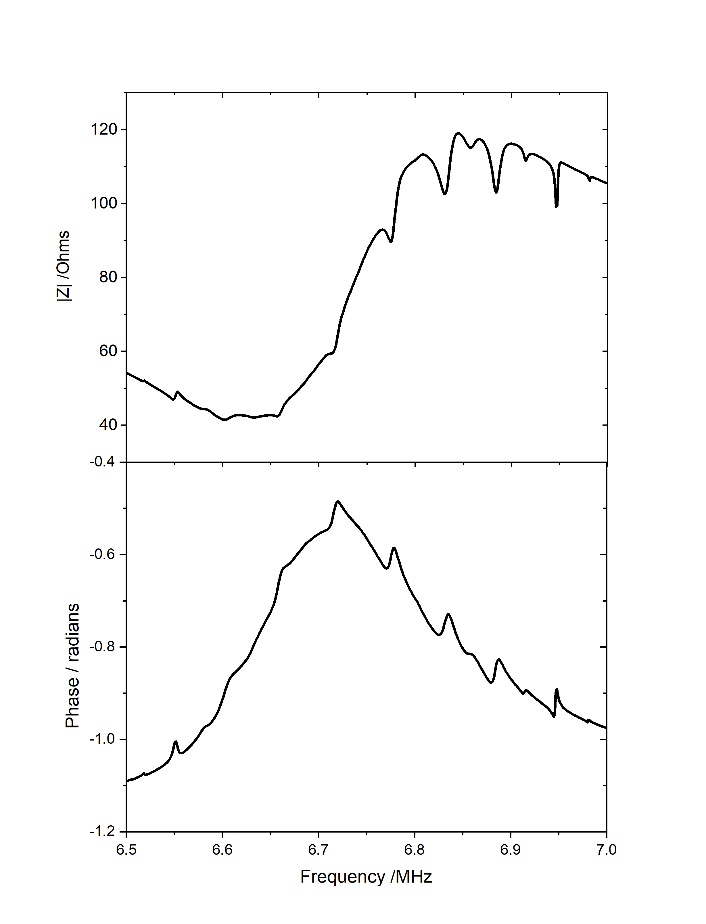


**Figure S4.** A transmission line calculation of the device assuming that the fraction of the resonating to non-resonating part of the cavity is 1/20.

The result shows impedance spectra that are broadly similar to those observed in our device. The sharp dips in |Z| and peaks in ⱷ, are the characteristic of the acoustically resonating part of the cavity and their position *and* width are indicative of the strong variations in acoustic pressure in the active part of the cavity as a function of frequency.

**Comparison of the calculated electrical impedance with experimental measurements.**

Figure S5 shows the impedance measurement from a simple device constructed with piezoelectric transducers in direct contact with the fluid (no PMMA walls). Sharp dips in |Z| and peaks in ⱷ similar to those predicted in Figure S4 are observed. Figure S6 shows the same data but where the broad background impedance of the transducers has been removed to emphasise the effects of the cavity resonance. Very sharp dips and peaks characteristic of resonance in the cavity are observed. These peaks are associated with the active layer in the device and their narrowness supports the assertion in this paper that large variations in the acoustic pressure will occur for small and subtle variations in the operating condition. It should be noted that the measurements obtained here were strongest near the optimum filling of the device and quite large variations in the measured impedance and strength of these resonance peaks was observed when the cell was under- or over-filled.

**
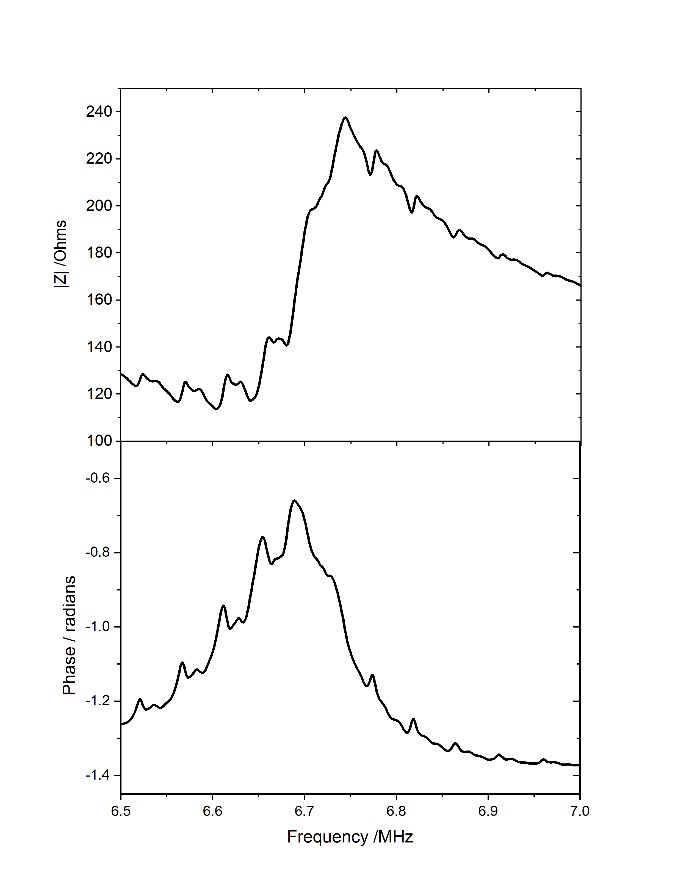
**

**Figure S5**. Impedance measurements for a simple (wall-less) device with transducers in direct contact with the fluid.


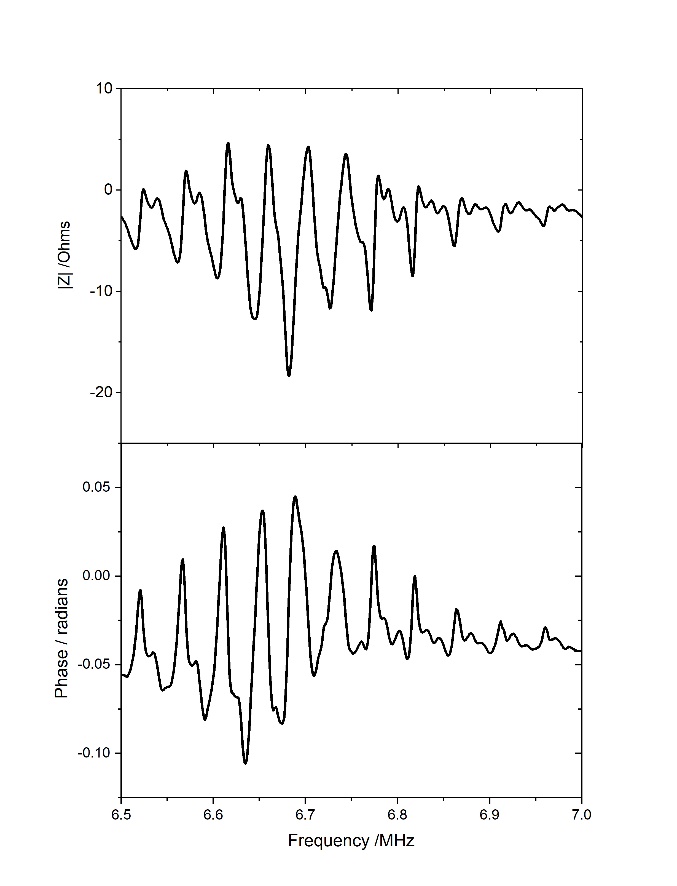


**Figure S6**. The same as the data shown in figure S5 except the smooth background from the transducer resonances has been removed to emphasise the effects from the cavity resonances.

Figure 7S shows the data from figure 8 in the paper for the measurement with level filling (+100µL) of the device. The weakness of the modulation is in agreement with that found in the work of Scholz et. al (reference 13) where it was found that the resonance in the cavity is confined to a small volume close to the bottom of the device. The spacing of the dips/peaks in |Z| and j correspond to that expected from the separation of the transducers in the device. Figure 8S shows the same data after the background contribution from the transducer resonances has been removed.


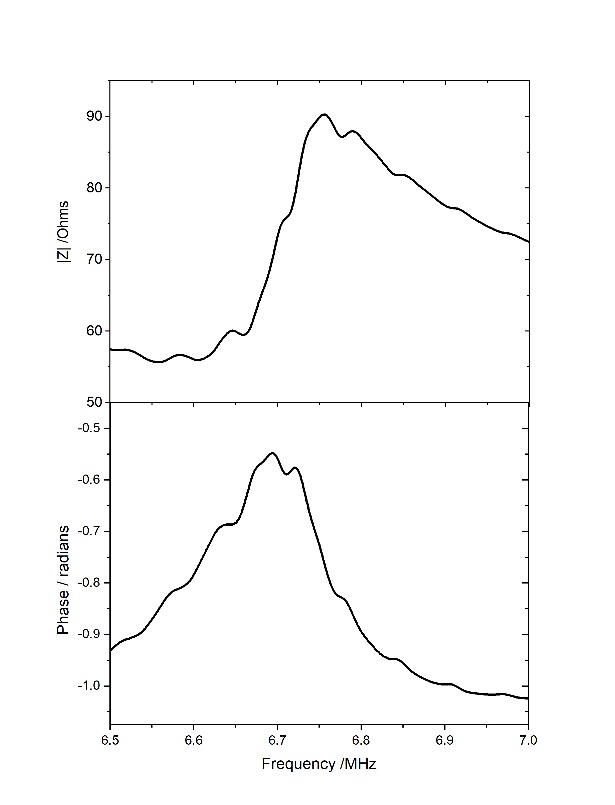


**Figure S7**. Impedance measurements for the device used in our work when filled to give a level surface (100µL added compared to our baseline measurement).


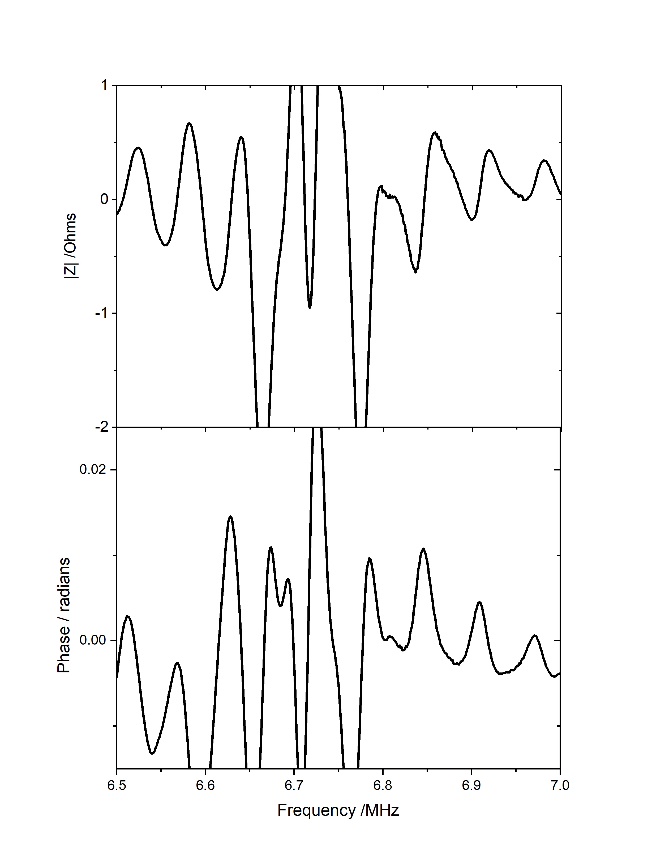


**Figure S8.** The data shown in figure 7 after the broad background contribution from the transducer resonances has been removed.

Again, the spacing of the fringes in the results is consistent with resonance in the cavity between the two transducers. There appears to be evidence of subtle variation in these peaks due to reflections, for example, from the PMMA walls (as seen in for example figure S4) and the resonance appears to be stronger at the higher frequencies. However, the sharpness of the peaks (especially as seen in ⱷ) confirms the highly resonant nature of the device in the active region. The full width half maximum of these peaks is ~ 15kHz. It may be seen from these observations how tuning steps for alignment of our cells in steps of 10 kHz may miss the maximum in the acoustic pressure. The wide variation in the impedance measurements with filling, the construction of the device, its temperature etc. will all lead to a difficulty producing reliable and consistent trapping forces on the cells and simple correlations with the applied voltage would not be expected.

1. C.R.P. Courtney, C.-K. Ong, B.W. Drinkwater, A.L.Bernassau, P.D. Wilcox and D.R.S. Cumming. Manipulation of particles in two dimensions using phase controllable ultrasonic standing waves. *Proc. R. Soc*. *A*. 2012. **468**, 337-360.

**Methods**

**Interpolated cell concentration values obtained from standard curves.**

Cell concentrations for each treatment after 24 hr incubation according to Calcein-AM signal values were obtained using standard cell-concentration curves.

The fluorescent standard curves were prepared with a range of concentrations from 2 X10^3^ cells ml^-1^ to 5 X10^5^ cells ml^-1^ (HeLa and HDF cells for each standard curve). The initial cell concentrations were calculated employing a dye-exclusion assay and with an automated cell counter. To obtain Calcein AM fluorescent values, the different cell concentrations were incubated at 37°C for 1h with 3µM Calcein-AM (490_Ex_/520_Em_) (Invitrogen, UK) solution in DMEM and fluorescent measurement were obtained prior to the different acoustic treatments and associated controls. The gain for the fluorescent readings was previously adjusted for HDF and HeLa plates, respectively, based on the well with the highest fluorescent value of each plate. Fluorescent readings for the standard curves were acquired with the CLARIOstar® microplate reader (BMG LABTECH, UK).

For each standard curve made in GraphPad Prism 8, we tried to fit a straight line with a nonlinear regression applying a Y=YIntercept +Slope*X model, where YIntercept= Y mean values for each voltage and control values at 5, 10 and 15 minutes. GraphPad Prism 8 also extrapolates slightly beyond the las X value (cell ml-1) entered, in this case beyond 5 X 10^5^ cell ml-1 when some X values are outside the entered range.

**Graph S1. Interpolated voltage and control mean values to obtain the cell concentration in HDF at 34°C.**

**Table S1. Voltages numerical data interpolated in the standard curve for HDF at 34°C at 5, 10 and 15 minutes, respectively. Outside output range refers that the X value (cell concentration) for that specific voltage is higher than the last X value represented in the Graph S1.**

|  | **[cell ml^-1^] (Interpolated)** | **A.U. (Entered)** |
| --- | --- | --- |
| 10 V (5’) | 739440.096 | 133362.333 |
| 8V (5’) | 567814.403 | 104934.333 |
| 6 V (5’) | 470110.274 | 88750.667 |
| 10 V (10’) | <Outside output range> | 136670.667 |
| 8 V (10’) | 502352.979 | 94091.333 |
| 6 V (10’) | 433387.962 | 82668 |
| 10 V (15’) | <Outside output range> | 152482.667 |
| 8 V (15’) | 736109.570 | 132810.667 |
| 6 V (15’) | 515665.018 | 96296.333 |

**Table S2. Voltages numerical data interpolated in the standard curve for HDF at 34°C at 5, 10 and 15 minutes, respectively. Outside output range refers that the X value (cell concentration) for that specific control is higher than the last X value represented in the Graph S1.**

| **Controls** | **[cell ml^-1^] (Interpolated)** | **A.U. (Entered)** |
| --- | --- | --- |
| 10 V (C5’) | <Outside output range> | 141288 |
| 8V (C5’) | 616926.635 | 109025 |
| 6 V (C5’) | 543733.395 | 98068 |
| 10 V (C10’) | <Outside output range> | 188445 |
| 8 V (C10’) | <Outside output range> | 144796 |
| 6 V (C10’) | 487059.903 | 89584 |
| 10 V (C15’) | <Outside output range> | 187435 |
| 8 V (C15’) | <Outside output range> | 124368 |
| 6 V (C15’) | 393499.210 | 75578 |

**Graph S2. Interpolated voltage and control mean values to obtain the cell concentration in HDF at 20°C.**

**Table S3. Voltages numerical data interpolated in the standard curve for HDF at 20°C at 5, 10 and 15 minutes, respectively. Outside output range refers that the X value (cell concentration) for that specific voltage is higher than the last X value represented in the Graph S2.**

|  | **[cell ml^-1^] (Interpolated)** | **A.U. (Entered)** |
| --- | --- | --- |
| 10 V (5’) | 572825.284 | 105764.333 |
| 8V (5’) | 729386.135 | 131697 |
| 6 V (5’) | 627037.382 | 114744 |
| 10 V (10’) | 643186.908 | 117419 |
| 8 V (10’) | 596034.317 | 109608.667 |
| 6 V (10’) | 608450.837 | 111665.333 |
| 10 V (15’) | 717909.407 | 129796 |
| 8 V (15’) | <Outside output range> | 139787.667 |
| 6 V (15’) | <Outside output range> | 153106.667 |

**Table S4. Voltages numerical data interpolated for the standard curve in HDF at 20°C at 5, 10 and 15 minutes, respectively. Outside output range refers that the X value (cell concentration) for that specific control is higher than the last X value represented in the Graph S2.**

| **Controls** | **[cell ml^-1^] (Interpolated)** | **A.U. (Entered)** |
| --- | --- | --- |
| 10 V (C5’) | 611837.582 | 109794.5 |
| 8V (C5’) | 662378.776 | 117684 |
| 6 V (C5’) | 647638.262 | 115383 |
| 10 V (C10’) | 664415.927 | 118002 |
| 8 V (C10’) | 595107.963 | 107183 |
| 6 V (C10’) | 585710.164 | 105716 |
| 10 V (C15’) | 674845.113 | 119630 |
| 8 V (C15’) | <Outside output range> | 147586 |
| 6 V (C15’) | 718397.215 | 126428 |

**Graph S3. Interpolated voltage and control mean values to obtain the cell concentration in HeLa at 34°C.**

**Table S5. Voltages numerical data interpolated in the standard curve for HeLa at 34°C at 5, 10 and 15 minutes, respectively.**

|  | **[cell ml^-1^] (Interpolated)** | **A.U. (Entered)** |
| --- | --- | --- |
| 10 V (5’) | 5974.294 | 1742 |
| 8V (5’) | 8453.762 | 2317.667 |
| 6 V (5’) | 3193.327 | 1096.333 |
| 10 V (10’) | 5567.989 | 1647.667 |
| 8 V (10’) | 11326.614 | 2984.667 |
| 6 V (10’) | 5397.139 | 1608 |
| 10 V (15’) | 5167.426 | 1554.667 |
| 8 V (15’) | 15172.877 | 3877.667 |
| 6 V (15’) | 4303.130 | 1354 |

**Table S6. Controls numerical data interpolated in the standard curve for HeLa at 34°C at 5, 10 and 15 minutes, respectively.**

| **Controls** | **[cell ml^-1^] (Interpolated)** | **A.U. (Entered)** |
| --- | --- | --- |
| 10 V (C5’) | 9729.593 | 2332.5 |
| 8V (C5’) | 1968.440 | 1145.5 |
| 6 V (C5’) | 2194.017 | 1180 |
| 10 V (C10’) | 17693.439 | 3550.5 |
| 8 V (C10’) | 4276.517 | 1498.5 |
| 6 V (C10’) | 1458.440 | 1067.5 |
| 10 V (C15’) | 5665.940 | 1711 |
| 8 V (C15’) | 13711.516 | 2941.5 |
| 6 V (C15’) | 1755.940 | 1113 |

**Graph S4. Interpolated voltage and control mean values to obtain the cell concentration in HeLa at 20°C.**

**Table S7. Voltages numerical data interpolated in the standard curve for HeLa at 20°C at 5, 10 and 15 minutes, respectively.**

|  | **[cell ml^-1^] (Interpolated)** | **A.U. (Entered)** |
| --- | --- | --- |
| 10 V (5’) | 3392.891 | 1142.667 |
| 8V (5’) | 3424.476 | 1150 |
| 6 V (5’) | 3029.656 | 1058.333 |
| 10 V (10’) | 4769.735 | 1462.333 |
| 8 V (10’) | 6799.826 | 1933.667 |
| 6 V (10’) | 6235.593 | 1802.667 |
| 10 V (15’) | 3747.511 | 1225 |
| 8 V (15’) | 8900.267 | 2421.333 |
| 6 V (15’) | 5582.346 | 1651 |

**Table S8. Controls numerical data interpolated in the standard curve for HeLa at 20°C at 5, 10 and 15 minutes, respectively.**

| **Controls** | **[cell ml^-1^] (Interpolated)** | **A.U. (Entered)** |
| --- | --- | --- |
| 10 V (C5’) | 1393.667 | 678.5 |
| 8V (C5’) | 8872.989 | 2415 |
| 6 V (C5’) | 2799.943 | 1005 |
| 10 V (C10’) | 2715.954 | 985.5 |
| 8 V (C10’) | 7208.285 | 2028.5 |
| 6 V (C10’) | 2834.4 | 1013 |
| 10 V (C15’) | 5186.090 | 1559 |
| 8 V (C15’) | 12936.761 | 3358.5 |
| 6 V (C15’) | 6217.647 | 1798.5 |
